# Capacity of Aqueous Solutions of the Ionic Liquid 1-Ethyl-3-methylimidazolium Acetate to Partially Depolymerize Lignin at Ambient Temperature and Pressure

**DOI:** 10.1021/acs.jafc.3c04047

**Published:** 2024-01-06

**Authors:** Carlos A. Pena, Eva Rodil, Héctor Rodríguez

**Affiliations:** CRETUS, Department of Chemical Engineering, Universidade de Santiago de Compostela, E-15782 Santiago de Compostela, Spain

**Keywords:** Indulin AT, ionic liquid, 2D-NMR, nanoparticles, vanillin, guaiacol

## Abstract

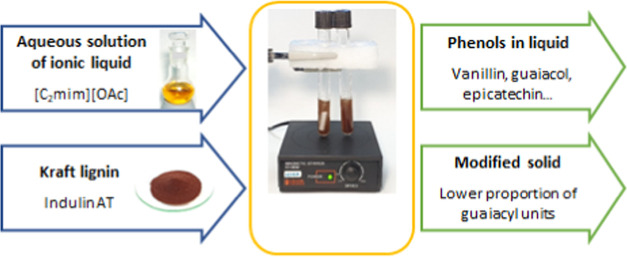

Lignin is a very attractive and abundant biopolymer with the potential to be a biorenewable source of a large number of value-added organic chemicals. The current state-of-the-art methods fail to provide efficient valorization of lignin in this regard without the involvement of harsh conditions and auxiliary substances that compromise the overall sustainability of the proposed processes. Making an original approach from the set of mildest temperature and pressure conditions, this work identifies and explores the capacity of an aqueous solution of the nonvolatile ionic liquid 1-ethyl-3-methylimidazolium acetate ([C_2_mim][OAc]) to partially depolymerize technical lignin (Indulin AT) by means of a treatment consisting in the simple contact at ambient temperature and pressure. Among a considerable number of valuable phenolic molecules that were identified in the resulting fluid, vanillin (yield of about 3 g/kg) and guaiacol (yield of about 1 g/kg) were the monophenolic compounds obtained in a higher concentration. The properties of the post-treatment solids recovered remain similar to those of the original lignin, although with a relatively lower abundance of guaiacyl units (in agreement with the generation of guaiacyl-derived phenolic molecules, such as vanillin and guaiacol). The assistance of the treatment with UV irradiation in the presence of nanoparticle catalysts does not lead to an improvement in the yields of phenolic compounds.

## 1 Introduction

In the frame of the UN Sustainable Development Agenda (https://sdgs.un.org/2030agenda), the substitution of fossil and nonrenewable resources by others with a more satisfactory (bio)renewable character in the industry is an unstoppable trend. In that race, an abundant and well geo-distributed bioresource called to play a remarkable role in the configuration of the next industrial platforms for the production of chemicals and materials is lignocellulosic biomass.^1^ Although the composition of this type of biomass is very variable depending on the species and many other factors, in general, it can be said that its three major components are cellulose, hemicellulose, and lignin.^1^ For instance, in woody biomass, these three biopolymers typically represent more than 95% in dry weight.^2^ The valorized exploitation of this bioresource has traditionally focused on the valorization of cellulose, for example, in the pulp and paper sector or in the production of cellulosic fuels. However, at present, there exists a generalized consensus about the fact that for the viability of biorefinery schemes based on lignocellulosic feedstocks, an integral valorization of the other major biopolymers will also be needed.^3^ In fact, lignin can represent as much as 40% on a dry basis of the lignocellulosic feedstock.^4^

Lignin is a three-dimensional heteromorphic polymer, mainly built through the random interlinkage of three repetitive units: *p*-hydroxyphenyl (commonly known as H-unit), guaiacyl (or G-unit), and syringyl (or S-unit).^4,5^ This attractive chemical composition has resulted in it being suggested as a resource of great potential in added-value applications, for example, in the fields of bioplastics, resins, lubricants, and aerogels.^6,7^ Nevertheless, lignin has been commonly regarded as a low-value byproduct or even as a residue due to the difficulty in transforming it into valuable substances in a sufficiently attractive manner for the chemical industry.^8^ For this reason, most of the implemented processes for lignin valorization rely on its utilization as a simple fuel.^9^

Most of the efforts made to develop innovative processes to obtain products from lignin through its depolymerization involve, unfortunately, the use of solvents with poor green credentials and/or high temperatures and pressures.^10−13^ One of the alternatives that has been explored in some works over the past decade is the use of ionic liquids (ILs), which have been credited with the potential to act as both solvents and catalysts in the depolymerization of lignin.^14,15^ As compared to other solvents of organic nature, ILs can certainly improve the sustainability credentials of the lignin depolymerization processes thanks to inherent characteristics such as their extremely low vapor pressure (resulting in a lack of contribution of the solvent to atmospheric pollution and also beneficial effects in terms of process safety and health of plant operators). However, to date, the published research works exploring ILs for the valorization of lignin through its depolymerization have involved relatively harsh conditions: temperatures higher than 100 °C, oxygen pressures, additional metal/acid catalysts, etc.^16^ Such is the case, in particular, of the works investigating, for this purpose, the IL 1-ethyl-3-methylimidazolium acetate ([C_2_mim][OAc]).^17−20^ This is an archetypal IL in the pretreatment and dissolution of lignocellulosic biomass, exhibiting a remarkably high lignin dissolution capacity,^16,18,21,22^ along with other appealing attributes: reasonably good thermal stability, liquid character down to far below room temperature, good biocompatibility, and low toxicity.^16,23−25^ Theoretical studies have evidenced a strong hydrogen-bond interaction between lignin model solutes and ILs like [C_2_mim][OAc], containing an anion with a remarkable hydrogen-bond acceptor character.^26^ Nevertheless, it has also been shown that both the [C_2_mim]^+^ cation and the [OAc]^−^ anion play an active role, for example, in the mechanisms of dissolution of lignin in this IL, with the two ions establishing key interactions with hydroxyl groups of the lignin structure.^27^

Given the potential contribution that a reduction of the severity of the conditions would have in the sustainability of the corresponding lignin valorization process, the present work aims at establishing a reference counterpoint by exploring the IL-assisted depolymerization of lignin to yield value-added chemicals using the mildest temperature and pressure conditions: ambient temperature and atmospheric pressure. In particular, aqueous solutions of [C_2_mim][OAc] will be investigated due to the benign attributes displayed by this IL—see above. The combination of the IL with water will enable the modulation of the lignin solubility capacity of the resulting solution^28^ while simultaneously mitigating potential problems of pumping and mass transfer that the relatively high viscosity of [C_2_mim][OAc] might pose if used in neat.^29^ Indulin AT, a popular pine kraft lignin with a low degree of sulfonation, was selected as the lignin substrate of reference. Complementarily, in view of the previous literature on the depolymerization of lignin for the production of valuable chemicals via nanoparticle-catalyzed photoreaction,^30,31^ the assistance of the [C_2_mim][OAc]-based treatment with UV irradiation in combination with nanoparticles (TiO_2_ or AgCl), and optionally in the presence of a well-known oxidizing agent such as H_2_O_2_,^32^ has also been explored.

## 2 Materials and Methods

### 2.1 Materials

Indulin AT, a technical pine Kraft lignin commercialized by MeadWestvaco, was used as received. Its degree of sulfonation was measured by X-ray spectrometry using an Oxford Instruments Lab-X3500S spectrometer, resulting in a sulfur content of 2.1%, which is lower than those of other popular technical lignins.

The IL [C_2_mim][OAc] was purchased from IoLiTec with a nominal purity >95% (although the certificate of analysis of the specific lot, supplied by the manufacturer, indicated an actual purity of 99%). Prior to use, it was subjected to a high vacuum (an absolute pressure of ca. 1 Pa) while magnetically stirred at ca. 70 °C for a minimum of 48 h for the elimination of potential volatile impurities. After this purification step, the preservation of the chemical identity of the IL, as well as the absence of organic impurities at relevant levels, was verified by ^1^H and ^13^C NMR spectroscopy (spectra available in Figures S1 and S2 in the Supporting Information). Its water content was lower than 0.04 wt %, as measured by Karl Fischer titration in a Metrohm 899 coulometer.

Titanium oxide (TiO_2_) nanoparticles were supplied by Aldrich with a nominal purity greater than 99.5%. A particle size range of 20–50 nm was ascertained by transmission electron microscopy (TEM) analysis. Their crystalline structure was identified as anatase by means of X-ray diffraction (XRD). Silver chloride (AgCl) nanoparticles were synthesized from the bulk solid (Sigma-Aldrich, 99%) following the procedure described by Rodríguez-Cabo et al.^33^ The range of their particle size, measured by TEM, was 5–20 nm, and the crystalline structure obtained by XRD was chlorargyrite. Both TEM photographs and XRD patterns for these nanoparticles are presented in Figure S3 in the Supporting Information, along with additional experimental details.

Hydrogen peroxide (Sigma-Aldrich, 30% w/w) was diluted to the desired concentration by the addition of water. Bidistilled water, produced by a Bibby Aquatron A4000D system, was used in all of the experiments.

### 2.2 Treatment of Lignin

Treatments of lignin were carried out in Pyrex tubes with an outside diameter of 16 mm and a wall thickness of 1.8 mm. A solid load of 5 g of Indulin AT per 100 g of treatment fluid was systematically used, as well as magnetic stirring, to facilitate an intimate contact between the lignin and the formulation. Two different concentrations of [C_2_mim][OAc] (in water) were used in the treatments: either 10% (w/w) or 70% (w/w). Pure water (0% ionic liquid) was also used to establish a convenient reference case. Lignin treatments with IL concentrations of 0 or 10% were performed with partial solubilization, while the concentration of 70% of [C_2_mim][OAc] enabled a complete dissolution of the lignin load. Treatment times of up to 6 h were investigated. All experiments were performed at room temperature (ca. 22 °C) and atmospheric pressure.

For the experiments with complete dissolution of lignin, after the corresponding treatment time, the addition of water (twice the volume of the treatment medium) was carried out to induce precipitation. Then, the solid materials were separated from the liquid phase by filtration using nylon filters with a pore diameter of 0.22 μm. Finally, the recovered solids were washed twice with water (10 mL each time). For the experiments with just partial solubilization of lignin, the precipitation step was skipped, while the remaining steps were applied as already described.

The assistance of ultraviolet (UV) irradiation or nanoparticles in the treatments of lignin was also explored. The UV light source was a low-pressure mercury vapor lamp manufactured by UVP, model Pen-Ray 3SC-9, with a maximum emission wavelength of 254 nm. The nanoparticles used were either TiO_2_ or AgCl (see Section 2.1). Supplementation with hydrogen peroxide was also explored, adding, in such cases, H_2_O_2_ to the media about 1 min before the addition of lignin for initiation of the treatments.

### 2.3 Characterization of the Post-Treatment Aqueous Phases

The aqueous phases obtained as filtrates after the lignin treatments were analyzed by high-performance liquid chromatography (HPLC) in an HP Series 1100 HPLC chromatograph equipped with an HP G1315A diode array detector. A Zorbax SB-C18 reversed-phase column (4.6 mm × 150 mm, 5 μm particle size) was used, together with a precolumn of the same kind, at 40 °C. The injection volume was 4 μL, and the flow rate of the mobile phase was 1 mL/min. The HPLC-grade solvents used were aqueous 0.1% formic acid (prepared from formic acid commercialized by Scharlau with nominal purity >98%) as solvent A and acetonitrile (Supelco, >99.9%) as solvent B. The elution gradient for solvent B was as follows: from minute 0 to 15, eluent B at 5%; from minute 15 to 50, a linear increase to 60%; from minute 50 to 55, a linear increase to 100%; and concluding with a column recondition step consisting in a linear decrease to 5% from minute 55 to 60, and remaining constant until minute 70. Phenolic compounds were identified by comparing their retention times and wavelengths of maximum absorbance peaks with several standards: benzyl phenyl ether (Aldrich, 98%), catechin hydrate (Sigma-Aldrich, 98%), chlorogenic acid (Sigma-Aldrich, >95%), *p*-coumaric acid (Sigma, ≥98%), epicatechin (Sigma-Aldrich, 97%), guaiacol (Sigma-Aldrich, 99%), 4-hydroxybenzoic acid (Sigma-Aldrich, 99%), isoeugenol (Aldrich, 98%), (±)-naringenin (Aldrich, ≥95%), quercetin dehydrate (Sigma-Aldrich, 98%), syringaldehyde (Aldrich, 98%), *trans*-cinnamic acid (Sigma-Aldrich, 99%), and vanillin (Sigma-Aldrich, 99%). The obtained concentrations were related to the original mass of lignin involved in the treatment with calculated overall uncertainties in the range of 0.02–0.07 g of phenolic compound per kg of lignin.

### 2.4 Characterization of the Post-Treatment Recovered Solids

The solid samples recovered by filtration in the treatments and also the raw Indulin AT as a reference were characterized by two-dimensional ^1^H–^13^C heteronuclear single quantum coherence nuclear magnetic resonance (2D ^1^H–^13^C HSQC NMR) spectroscopy, thermogravimetric analysis (TGA), and differential scanning calorimetry (DSC), following the procedures described in the next paragraphs.

The 2-D NMR analyses started with the direct dissolution of the samples in 5 mm diameter NMR tubes with deuterated dimethyl sulfoxide (DMSO-*d*_6_, supplied by Sigma-Aldrich with a purity of 99% and an atomic deuteration level of 99.5 D%). The procedure followed was similar to what has been previously reported in the literature by a number of authors.^34−36^ In particular, herein an 11.7 T Bruker DRX-500 NMR spectrometer operating at a frequency of 500 MHz for ^1^H and equipped with a BBI probe with PFG gradient on the *z*-axis was used to record the spectra at 300 K. The 2D ^1^H–^13^C HSQC NMR spectra were measured for each sample with the center at 4.7 and 100 ppm, and spectral widths of 11 and 240 ppm, for the ^1^H and ^13^C dimensions, respectively. The number of collected complex points was 2048 for the ^1^H dimension and 256 for the ^13^C dimension. The spectra were acquired with a recycle delay (d_1_) of 5 and 64 scans per t_1_ increment. The nominal value of ^1^*J*_CH_ used for the INEPT periods was 140 Hz. The processing of the spectra was carried out with the software Mestrenova v.14.0 (Mestrelab Research Inc.). Prior to the 2D Fourier transformation, the FID files were apodized with a 90°-shifted sine-bell function in both dimensions and with line broadening of 8 and 14 Hz for the ^1^H and ^13^C dimensions, respectively. Then, they were zero-filled to 2048 and 512 points in the ^1^H and ^13^C dimensions, and subsequently, the Fourier transformation was applied in both dimensions. Next, the spectra were phase-corrected and baseline-corrected in both dimensions and finally subjected to a processing operation of T_1_ noise reduction.

The TGA analyses were carried out in a TA Instruments TGA Q500 thermogravimetric analyzer with a weight precision of 0.01%, using flow rates of 40 and 60 mL/min of nitrogen gas (Nippon Gases, 99.999%) as balance purge gas and sample purge gas, respectively. An amount of ca. 10–15 mg of each sample was placed in an open platinum pan, automatically introduced by the apparatus into the furnace chamber. The thermal program consisted of fast heating from room temperature to 105 °C, followed by a 15-min isotherm at this temperature to eliminate traces of humidity, and continuing with a heating ramp at a rate of 10 °C/min up to 800 °C. The software Universal Analysis 2000 by TA Instruments was used to process the thermogram. An uncertainty of 2 K was estimated for the reported temperatures.

The DSC analyses were performed in a TA Instruments DSC Q2000 differential scanning calorimeter with an RCS 90 refrigerated cooling system attached and using a flow rate of 50 mL/min of nitrogen gas as purge gas in the measurement chamber. For each run, ca. 5–10 mg of sample was placed in a 40-μL aluminum pan hermetically closed with a lid of the same material. An analogous empty pan with a lid was used as a reference. The thermal procedure comprised three cycles, each of them consisting of a heating ramp at a rate of 10 °C/min up to 200 °C, a 5 min isotherm, a cooling ramp at a rate of −10 °C/min down to 0 °C, and another 5 min isotherm. Essential overlapping of the thermograms for the second and third cycles was verified prior to validating the analyses. Glass transition temperatures (evaluated at the midpoint) were determined from the signal of the heating ramp of the third cycle with the corresponding function of Universal Analysis 2000, with an estimated uncertainty of 1 K.

## 3 Results and Discussion

### 3.1 Aqueous Solutions of [C_2_mim][OAc] as Treatment Media

Initial treatments of Indulin AT were performed with aqueous solutions of [C_2_mim][OAc] for two different IL concentrations (10% and 70%) and different treatment times (up to 6 h). A number of lignin-derived compounds were identified in the post-treatment aqueous phases, with the full list and corresponding yields (interpreted as mass of compound per unit mass of the original Indulin AT) being given in Table S1 in the Supporting Information. Apart from the polyphenolic compound epicatechin, the two (monophenolic) compounds obtained in a higher yield are vanillin and guaiacol, in line with what has been typically reported in the depolymerization of kraft lignins.^10,37−41^ These two substances are likely produced by the cleavage of β-O-4 bonds in the lignin polymeric structure by the action of the IL,^42,43^ and further analysis will specifically focus on them. From a mechanistic perspective, and according to a suggestion from the literature,^44^ the cleavage of these β-O-4 bonds may take place heterolytically via a six-membered transition state, with participation of both cation and anion of the IL. The cation would form an adduct that would polarize the ether bond, leading to an increased negative partial charge on the oxygen atom, which in turn would reduce the energy required for the heterolytic cleavage of the bond (with the anion being involved in this latter step).

**Figure 1:**
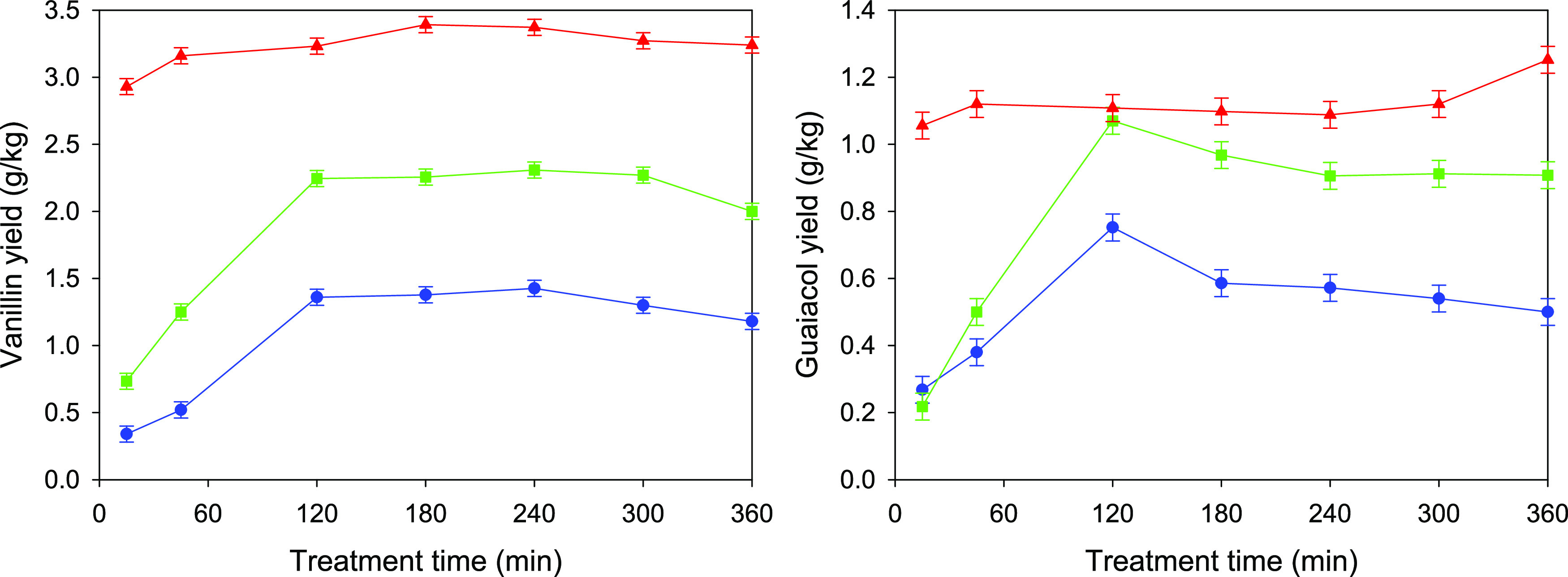
Vanillin (left) and guaiacol (right) yields, represented as g of phenol per kg of original Indulin AT, for treatments with pure water (blue circles) and aqueous solutions with [C_2_mim][OAc] concentrations of 10% (green squares) and 70% (red triangles).

Figure 1 shows the evolution of vanillin and guaiacol yields with the treatment time, not only for the treatments with the aqueous solutions of [C_2_mim][OAc] but also for analogous ones with plain water for comparison purposes. Even in the case of using just water, non-negligible yields of vanillin and guaiacol were obtained, probably due to the presence of these compounds or easily hydrolyzable fractions of low molecular weight in the raw Indulin AT. A significant increase in these yields, however, was found with increasing the presence of the IL in the treatment fluid for any given treatment time. For the treatments with water and with aqueous 10% IL solution, the evolution with the treatment time is similar: an initial increase up to 2 h of treatment, followed by a stabilization and small decrease at higher treatment times in the case of vanillin or directly by an appreciable decrease in the case of guaiacol for treatment times of ≥3 h. This decrease observed for the longer treatment times may be a result of the degradation/oxidation that phenols are known to undergo in nonoptimal atmospheres.^38,45,46^ Such a decrease is not observed for the treatments with an aqueous 70% IL solution, for which both the vanillin and guaiacol yields remain reasonably stable, and even with a slight increase at higher times in the case of guaiacol, suggesting the preference for a 6-h treatment in these conditions. The high concentration of IL may provide, in this case, a protective environment against the mentioned degradation/oxidation phenomena.

The yields reported in Figure 1 are clearly lower than the highest yields available in the literature. Focusing, for instance, on the particular case of vanillin, yields in the range of 25–100 g of vanillin per kg of lignin have been reported.^12,37,39,47^ Nevertheless, it must be noted that the latter yields were achieved through treatments involving stages with harsh conditions: very high pH (close to 14), temperatures over 150 °C, pressurization with molecular oxygen, and organic solvents, such as nitrobenzene. Different works,^10,38,48^ in the quest for greener approaches, avoided the use of highly toxic organic solvents; and, although they maintained strongly alkaline conditions, O_2_ pressurization, and temperatures never lower than 120 °C, their reported yields of vanillin decreased significantly, down to the range of 2.5–13 g of vanillin per kg of lignin. In this range also lie the yields reported in the present work, which in turn have been achieved by means of treatments at ambient pressure and temperature with the only involvement of a nonflammable IL with low toxicity. Thus, the results reported herein can be taken as a reference point of the yields that can be obtained at the mildest temperature and pressure conditions thanks to the lignin depolymerization capacity of an IL with attractive characteristics, namely, [C_2_mim][OAc]. Interestingly, Ogawa and Miyafuji reported a remarkable yield of 14 g of vanillin per kg of lignin (from Japanese cedar or Japanese beech) using the higher-melting, more toxic, and more viscous IL 1-ethyl-3-methylimidazolium chloride ([C_2_mim]Cl) at a moderately high temperature of 120 °C without pressurization;^46^ thus constituting an illustrative example of how the yield can be improved through partial sacrifices in the mildness of the treatment conditions.

**Figure 2:**
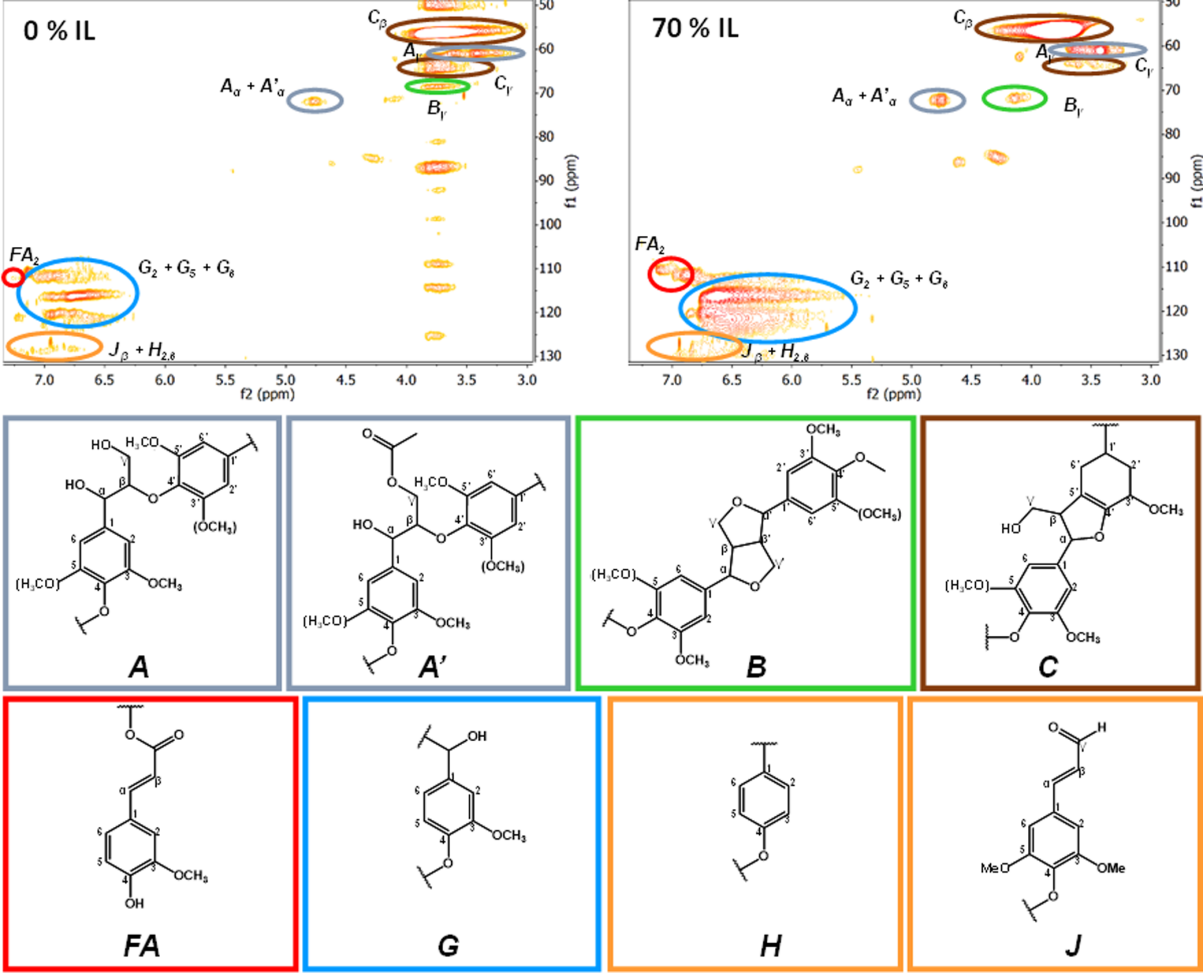
2D ^1^H–^13^C HSQC NMR spectra of the solid recovered after a 6-h treatment of Indulin AT with pure water (top left) or with a 70% solution of [C_2_mim][OAc] (top right). The colored ellipses in the spectra indicate signals associated with the different generic structures shown in the bottom part of the figure with frames of the same color.

**Figure 3:**
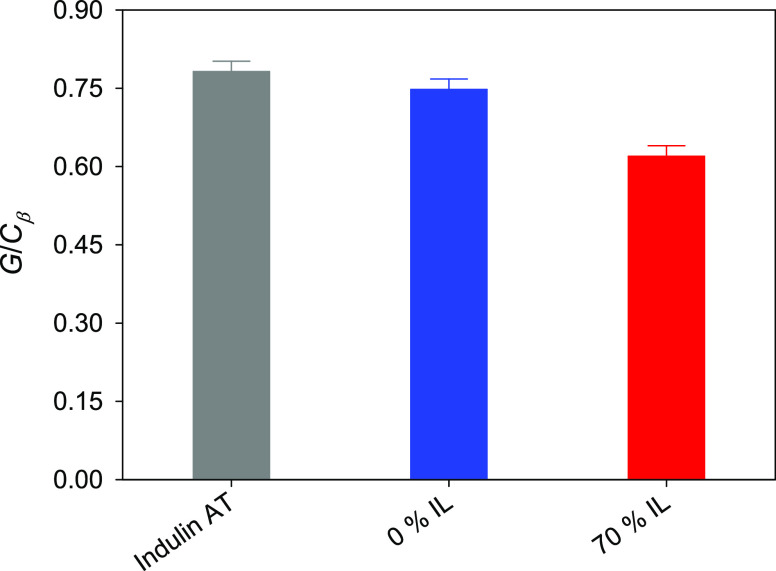
*G*/*C*_β_ ratio for raw Indulin AT and the solid recovered after the 6-h treatment with water (“0% IL”) or a 70% solution of [C_2_mim][OAc] (“70% IL”).

Regarding the solid phases recovered after treatment, the HSQC NMR spectra for the samples obtained in the treatment with pure water and in the treatment with the 70% IL solution (treatment time: 6 h) are shown in Figure 2. This figure also includes the chemical structures associated with the representative signal regions in the spectra. Structures *FA* and *G* are specifically related to lignin G-units.^34,49−52^ Since the absolute values of the integration of the areas under the signals are sensitive to the actual concentration of the solute in the preparation of the sample for NMR analysis and to subsequent processing (e.g., phase correction of the Fourier-transformed signal), area ratios have been used in order to establish valid comparisons.^5,12,40,53^ In particular, area ratios related to the biggest area (the one corresponding to the *C*_β_ region, see Figure 2) were calculated. Table S2 in the Supporting Information lists the numerical values (with an estimated uncertainty of 0.01) of the so-calculated ratios for the two selected recovered solids plus those obtained directly from the analysis of raw Indulin AT (see the spectrum in Figure S4 in the Supporting Information). All ratios were found to be very small in comparison to the *G*/*C*_β_ ratio (i.e., the ratio between the areas of the *G* region and the *C*_β_ region). Therefore, this *G*/*C*_β_ ratio was taken as a good indicator of the relative abundance of G-units in the recovered lignin samples after the investigated treatments. In Figure 3, it can be seen how the *G*/*C*_β_ ratio is considerably lower for the solid sample recovered after the treatment with the 70% IL solution than in the case of the solid sample recovered after the treatment with plain water. The latter, at the same time, is just slightly lower than that for untreated Indulin AT. This evolution is in good agreement with the trend followed by the yields of vanillin and guaiacol calculated from their concentrations in the post-treatment aqueous phases: the higher yields of these phenolic compounds (derived from lignin G-units) in the aqueous phase correspond with the stronger relative diminution of G-units (i.e., lower *G*/*C*_β_ ratio) in the solid phase. Since this has not been accompanied by an increase in the signals associated with other lignin structures (condensation products proposed by Yang et al.),^40^ it can be guessed that the relative diminution of G-units should be connected with the depolymerization of lignin for subsequent transformation into small phenolic compounds.

Although the results herein reported are attractive, it must be noted that the viability of the investigated treatment approach in a practical application would be conditioned by the efficient recyclability of the IL. To avoid the buildup of lignin fragments and the small lignin-derived molecules, these have to be effectively removed. For their recovery by precipitation upon the addition of an antisolvent such as water, the excessively large amount of water necessary to carry out a complete precipitation would impose a significant energy penalty in the step of eliminating the water to get the IL ready for its recycling. Still, it is interesting to point out that the fact of using in the investigated treatments the IL in solution (at a concentration not higher than 70%), and not the pure IL, would avoid the most costly part of the IL recovery step if performed by vacuum distillation.^54^ In any case, exploration of alternative strategies to improve the recyclability of the IL in this kind of process will be very welcome toward the industrial feasibility of the proposed lignin depolymerization approaches.

### 3.2 Treatments Assisted by UV-Irradiation Photoreaction Catalyzed by Nanoparticles

**Figure 4:**
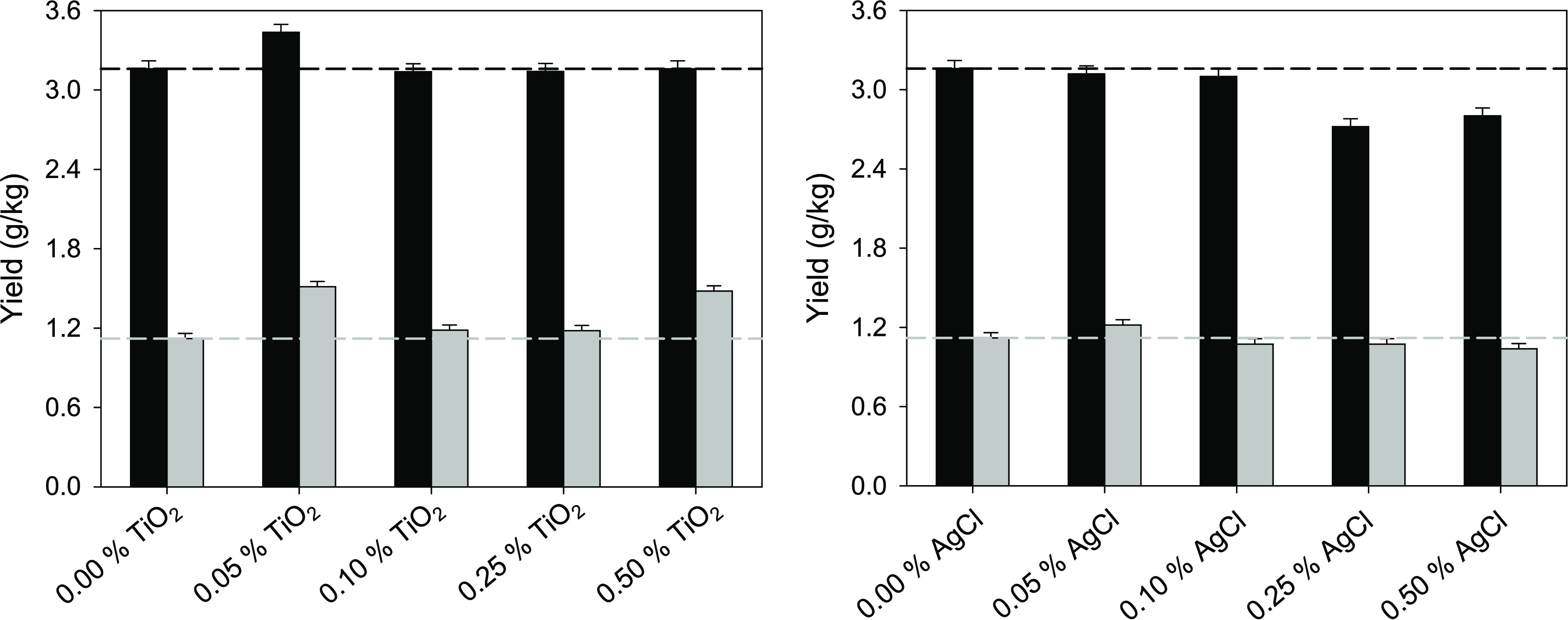
Yields of vanillin (black bars) and guaiacol (gray bars) as a function of the concentration of TiO_2_ nanoparticles (left) or AgCl nanoparticles (right) in 45-min treatments of Indulin AT with a 70% solution of [C_2_mim][OAc] assisted by UV irradiation. The horizontal dashed lines (black for vanillin and gray for guaiacol) indicate the yields obtained in equivalent treatments with neither UV irradiation nor nanoparticles.

With the purpose of enhancing the performance of the treatments investigated in Section 3.1, the deconstruction of lignin through the photoreaction induced by UV light with TiO_2_ or AgCl nanoparticles as catalysts was investigated. Since an excessively high concentration of nanoparticles can cause a shielding effect on UV irradiation,^55^ the first variable to explore was the concentration of nanoparticles. Using the 70% IL solution as treatment fluid of reference, four concentrations of nanoparticles were tested in quick 45-min treatments under UV irradiation, namely, 0.05, 0.10, 0.25, and 0.50% (w/w). Focusing on the yield of the two major phenolic compounds identified in the post-treatment aqueous phases (vanillin and guaiacol), Figure 4 shows that the concentration of 0.05% of TiO_2_ nanoparticles leads to a simultaneous improvement of the yields of these two phenols with respect to the case of using UV irradiation without nanoparticles (which, in turn, provides results equivalent to the analogous treatment without irradiation). Treatments with the other concentrations of TiO_2_ nanoparticles tested do not lead to such a simultaneous improvement of both yields. Regarding the AgCl nanoparticles, no significant improvement in the yields of the phenols was detected at any concentration, and even a clear decrease in the yield of vanillin was observed for the highest concentrations (≥0.25%). Therefore, the AgCl nanoparticles were discarded, and a concentration of 0.05% of TiO_2_ nanoparticles was selected for further experiments.

**Figure 5:**
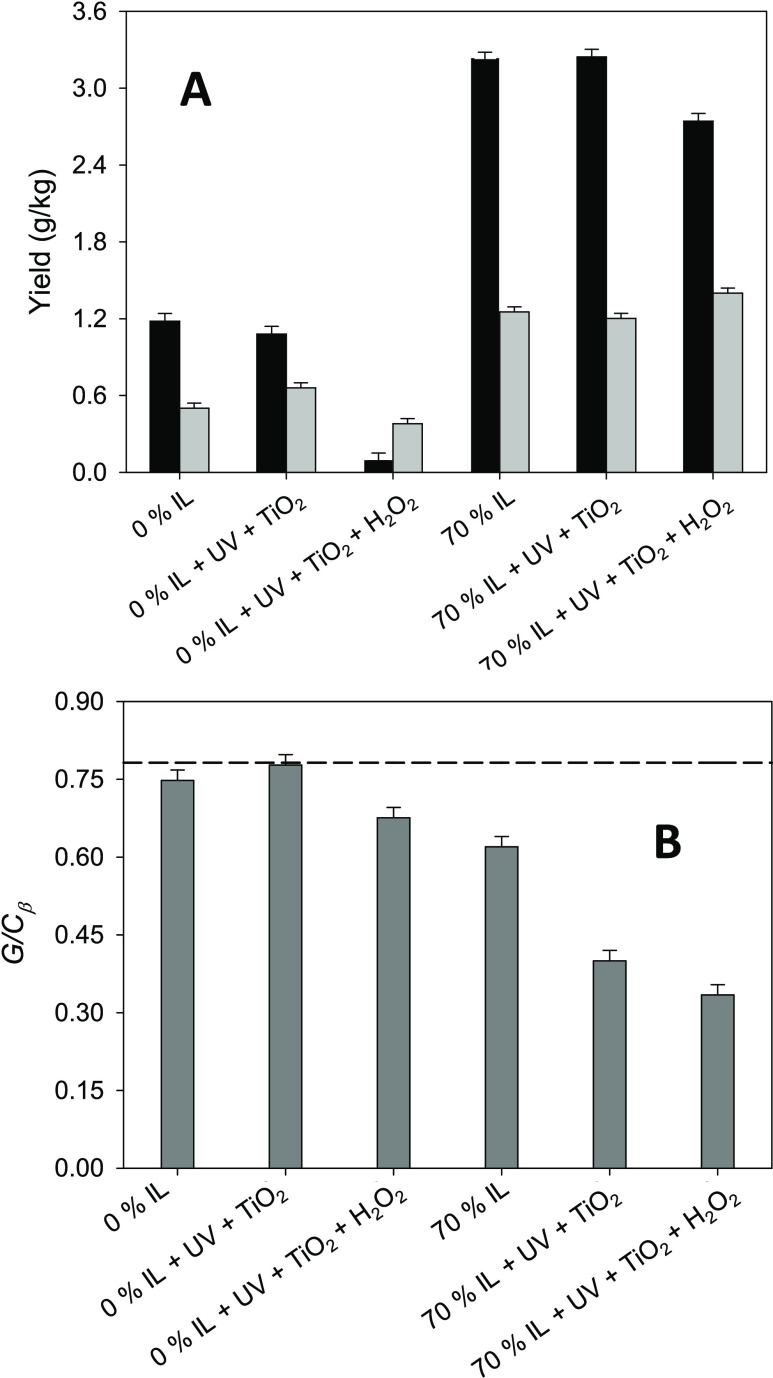
(A) Yields of vanillin (black bars) and guaiacol (light gray bars) for 6-h treatments of Indulin AT with water (0% IL) or a 70% solution of [C_2_mim][OAc] assisted by UV irradiation, with 0.05% TiO_2_ nanoparticles and, in treatments with a preactivation of nanoparticles, a 5 mM concentration of H_2_O_2_. (B) The corresponding *G*/*C*_β_ ratios (dark gray bars) of the recovered solids, with the *G*/*C*_β_ ratio for raw Indulin AT represented by the horizontal dashed line.

Figure 5A shows the effect of applying UV irradiation and a 0.05% concentration of TiO_2_ nanoparticles in the 6-h treatment of Indulin AT with either water (“0% IL”) or a 70% solution of [C_2_mim][OAc]. Disappointingly, no significant variation was observed in the yields of vanillin or guaiacol. In trying to boost the catalytic activity of the TiO_2_ nanoparticles, hydrogen peroxide (at a concentration of 5 mM) was investigated as an additive. However, these experiments involving H_2_O_2_ actually led to lower yields of the phenolic compounds, especially in the case of vanillin. Figure 5B presents the corresponding *G*/*C*_β_ ratios of the post-treatment recovered solids (values calculated from the NMR spectra shown in Figures 2 and S5 and numerically listed in Table S2 in the Supporting Information), which can be analyzed together with the yields shown in Figure 5A. It is observed that, for most of the UV-assisted experiments (the exception being the treatment with water in the absence of H_2_O_2_), a decrease in the *G*/*C*_β_ ratio value occurs without leading to a higher yield of vanillin and guaiacol, which are phenolic compounds derived from the lignin G-units. A plausible explanation may be that part of the G-units that leave the solid substrate do not end up transformed into the phenolic compounds of interest due to a certain oxidizing character of the TiO_2_ nanoparticles,^56^ and/or, even more notoriously, of the strong oxidizing character of H_2_O_2_ (masking its potential effect as activator of the nanoparticles to boost their catalytic activity). Instead, fully oxidized compounds of low molecular weight might have been produced, such as CO_2_,^56^ leaving the system, since neither an increase of other identified compounds nor new compounds were detected by HPLC in the analysis of the post-treatment aqueous phase of those experiments.

Finally, it is worth mentioning that the percentage of solid recovered after any of the treatments lied consistently within the range of 80–90%.

### 3.3 Thermal Characterization of the Recovered Solids

**Figure 6:**
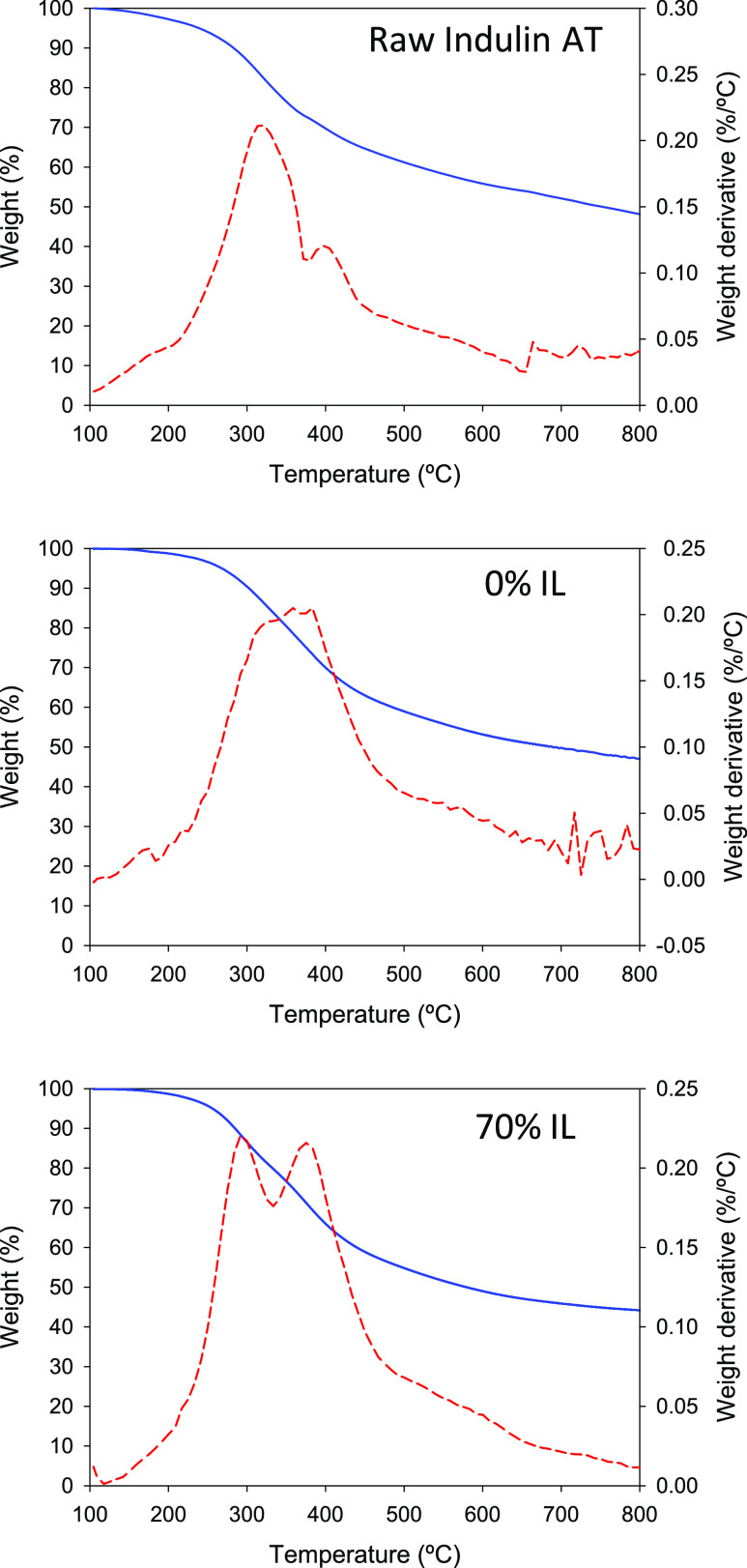
TGA thermograms (solid blue lines) and the corresponding derivative curves (dashed red lines) for raw Indulin AT (top plot) and for the solids recovered after the 6-h treatments with water (central plot) or with a 70% IL solution (bottom plot).

Thermal analysis of the treated lignin can provide complementary information about the mode of action of the treatment fluids investigated. For example, the thermal stability of lignin is highly influenced by its internal structure.^57^Figure 6 shows representative TGA thermograms of raw Indulin AT and the recovered solids from the treatments with plain water and the 70% solution of [C_2_mim][OAc], exhibiting behavior similar to that reported by Tan et al.:^58^ the first stage, up to ca. 200 °C, with a slight weight loss due to dehydration and vaporization of volatiles; the second stage, in the range of 200–350 °C, where the decomposition of lignin polymeric moieties with low molecular weight occurs, releasing CO, CO_2_, and H_2_O from cleavage of the side chains of lignin structures; and the third stage, above 350 °C, associated with the decomposition of aromatic rings, yielding volatile decomposition products.^58,59^ From the derivative curve of the TGA thermogram for raw Indulin AT, two maxima of decomposition rate can be identified, located respectively in the regions of the second stage and third stage described above. The clearly bigger size of the left (lower-temperature) peak may be indicative of a relatively high content of low molar mass polymeric moieties in the raw Indulin AT investigated and may explain in part the yields of small phenolic compounds obtained with only water (see, for instance, Figure 1). Interestingly, in the derivative curve of the thermogram for the solid recovered from the treatment with water (“0% IL”), only one peak was observed, although it might be the composed peak of the two above-mentioned peaks overlapping largely, for example, due to a more prolonged decomposition of low molar mass polymeric chains during the course of the dynamic TGA experiment. In the derivative curve of the thermogram of the solid recovered from the treatment with the aqueous 70% IL solution, again, the two peaks can be observed, although this time, their sizes are much more balanced than in the case of raw Indulin AT, likely indicating a relatively higher contribution of the aromatic rings to the total lignin with respect to raw Indulin AT. Complementarily, the large residue observed for all three thermograms in Figure 6 at 800 °C (the temperature at which the TGA runs were stopped) invites one to think that the thermal decomposition of the aromatic rings occurs to a limited extent. The numerical values of these solid residues at 800 °C, together with those of the temperatures of all of the maxima of the rate of decomposition (*T*_max_) for the thermograms in Figure 6, are summarized in Table 1. Additionally, this table includes the same information obtained from the TGA thermograms of the solids recovered from the treatments involving UV irradiation plus TiO_2_ nanoparticles and optionally H_2_O_2_ (see the thermograms in Figure S6 in the Supporting Information). A small reduction in the *T*_max_ values can be observed in some cases, but in general, it can be said that the influence of those elements on the thermal behavior of the solids recovered is rather limited.

**Table 1:** Numerical Values of Temperature(s) of Maxima of Decomposition Rate (*T*_max_), Percentage Residue at 800 °C, and Glass Transition Temperature (*T*_g_) for Raw Indulin AT and for the Recovered Solid Samples after Different 6-h Treatments

treatment	*T*_max_ (°C)	residue at 800 °C (%)	*T*_g_ (°C)
none (raw Indulin AT)	320, 405	45	154
water (0% IL)	357	48	163
water + UV + TiO_2_	338	52	163
water + UV + TiO_2_ + H_2_O_2_	335	48	163
70% IL solution	294, 377	44	152
70% IL solution + UV + TiO_2_	268, 372	41	162
70% IL solution + UV + TiO_2_ + H_2_O_2_	288, 379	43	161

By DSC analysis, the glass transition temperature (*T*_g_) of the recovered solid samples in the different treatments was found to be practically the same as that of the original Indulin AT (154 °C) or slightly higher (up to 163 °C). The numerical *T*_g_ values are detailed in Table 1, whereas the processed DSC thermograms from which they were obtained are shown in Figures S7 and S8 in the Supporting Information. The *T*_g_ for raw Indulin AT is very close to that of 157 °C reported by Li and McDonald,^60^ especially if taking into account that the glass transition of lignin is highly influenced by factors such as the water content of the sample.^61,62^ Even though efforts were made to maintain a very low and approximately constant water content for all of our samples, part of the variation observed in Table 1 for *T*_g_ may be due to this factor. In any case, it is clear that the treatments investigated in this work do not imply a relevant modification of the glass transition behavior of the lignin material.

## Supporting Information Available

^1^H and ^13^C NMR spectra of the purified ionic liquid; microscopy photographs and XRPD pattern of the nanoparticles; yield of phenolic compounds in the post-treatment aqueous phase, as determined from HPLC analyses; 2D ^1^H–^13^C HSQC NMR data of raw Indulin AT and the recovered solid samples; and TGA and DSC thermograms (PDF)
