# Supplementary material for: Capacity of Aqueous Solutions of the Ionic Liquid 1-Ethyl-3-methylimidazolium Acetate to Partially Depolymerize Lignin at Ambient Temperature and Pressure
Source: J Agric Food Chem. 2024 Jan 6;72(2):1136–45. doi: 10.1021/acs.jafc.3c04047 (PMC10797632; doi:10.1021/acs.jafc.3c04047)
Supplement: Supplementary file 1 — jf3c04047_si_001.pdf [file jf3c04047_si_001.pdf]

SUPPORTING INFORMATION

Capacity of aqueous solutions of the ionic liquid 1-ethyl-3-methylimidazolium acetate to partially depolymerize lignin at ambient temperature and pressure

Carlos A. Pena[ǂ], Eva Rodil[ǂ], and Héctor Rodríguez[ǂ,]*

[ǂ] *CRETUS, Department of Chemical Engineering, Universidade de Santiago de Compostela, E-15782, Santiago de Compostela, Spain*

* Corresponding author. Phone: +34 881816804. E-mail: hector.rodriguez@usc.es

Contents:

- ^{1}H and ^{13}C NMR spectra of the purified ionic liquid S2
- TEM photographs and XRD pattern of the nanoparticles S4
- Yield of phenolic compounds in the post-treatment aqueous phase, as determined from HPLC analyses S5
- 2D ^{1}H-^{13}C HSQC NMR data of raw Indulin AT and the recovered solid samples S6
- TGA and DSC thermograms S8

^{1}H and ^{13}C NMR spectra of the purified ionic liquid

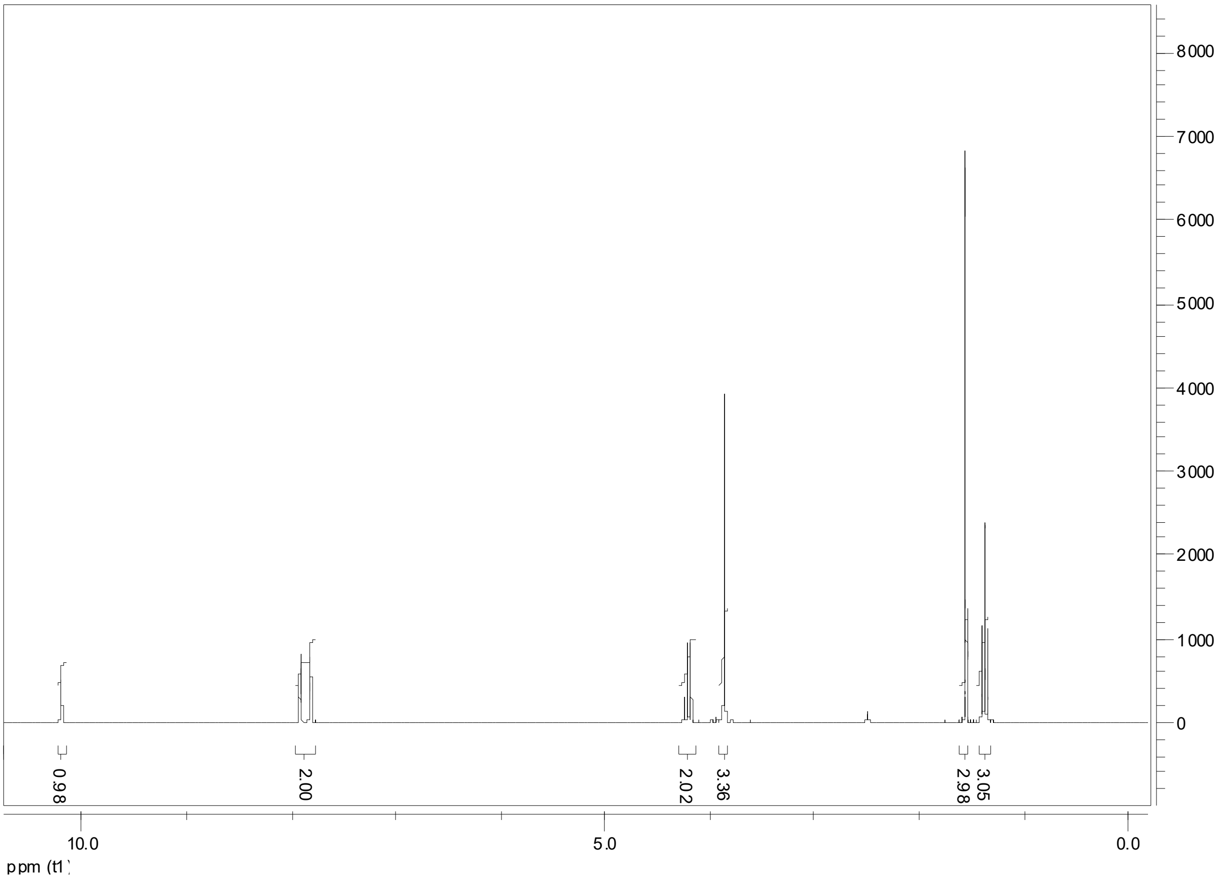

Figure S1. ^{1}H NMR spectrum of [C_2mim][OAc]. δ_H (DMSO-d_6, 300 MHz): 1.39 (t, J = 7.3 Hz, 3H, $NCH_2C\underline{H_3}$), 1.56 (s, 3H, CH_3COO), 3.87 (s, 3H, NCH_3), 4.22 (q, J = 7.3 Hz, 2H, NCH_2), 7.78-7.96 (unresolved, 2H, C(5)H and C(4)H), 10.20 (s, 1H, C(2)H). The peak at 2.50 ppm corresponds to the residual signal of the deuterated solvent.

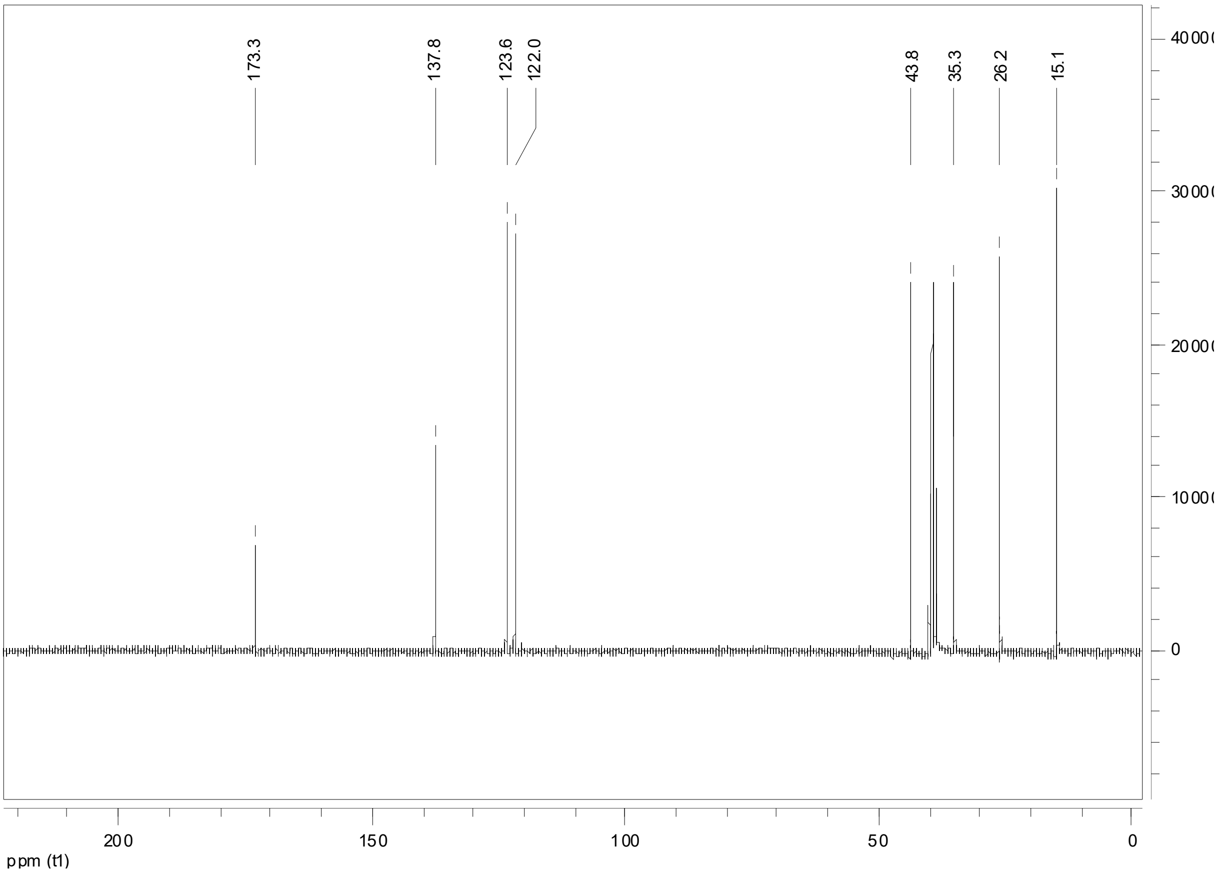

Figure S2. ^{13}C NMR spectrum of [C$_2$mim][OAc]. δ_C (DMSO-d_6, 75.4 MHz): 15.1 (NCH$_2$<u>C</u>H$_3$), 26.2 (<u>C</u>H$_3$COO), 35.3 (NCH$_3$), 43.8 (NCH$_2$), 122.0 (C(4)H), 123.6 (C(5)H), 137.8 (C(2)H), 173.3 (CH$_3$<u>C</u>OO). The multiplet at 40 ppm corresponds to the signal of the deuterated solvent.

TEM photographs and XRD pattern of the nanoparticles

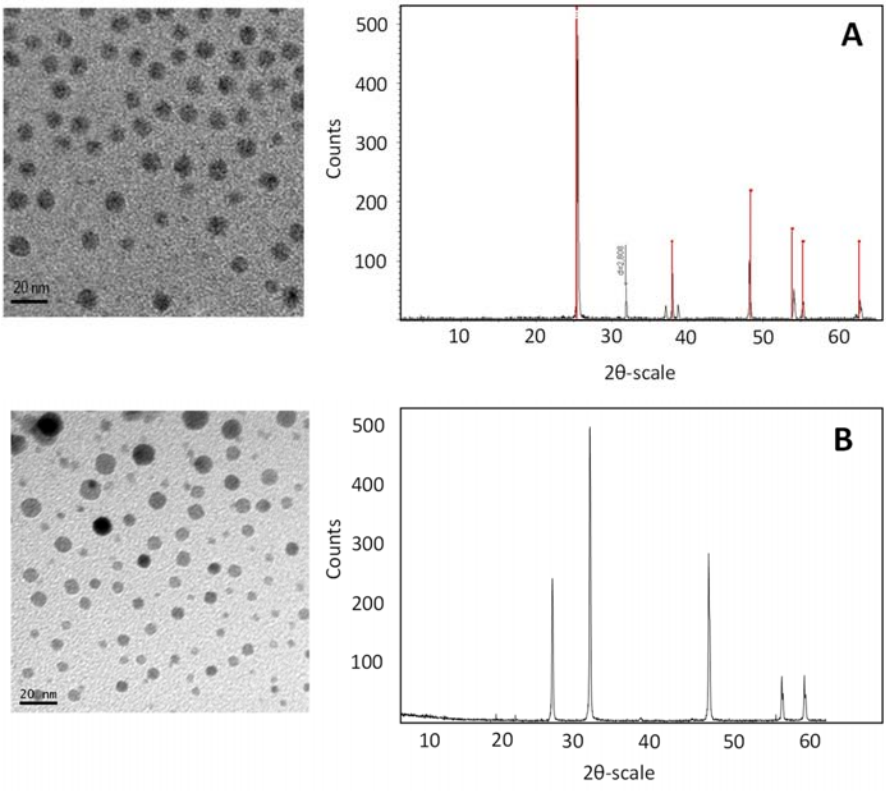

Figure S3. TEM photographs (left column) and XRD patterns (right column) for TiO_2 nanoparticles (A) and AgCl nanoparticles (B).

- Additional experimental details:
  - TEM images were performed using a Philips CM-12 microscope with a Mega View Docu-II camera and an IMAX image analysis software SIS NT.
  - XRD patterns were acquired in an X-ray Philips powder diffractometer operated by a PW 1710 control unit, with a Cu-Kα X-ray source (λ = 1.54 Å).

Yield of phenolic compounds in the post-treatment aqueous phase, as determined from HPLC analyses

Table S1. Yield of compounds (expressed in g of compound per kg of lignin) identified in the aqueous phase of the treatments, with different concentrations of the ionic liquid in the treatment fluid and different treatment times. Benzyl phenyl ether, catechin hydrate, chlorogenic acid, and 4-hydroxybenzoic acid were also included in the list of standards scrutinized, but no significant concentration greater than 0.01 g/kg could be detected for them in any of the treatments. The letter (H, G, or S) in parenthesis next to the name of each compound indicates the type of lignin unit (p-hydroxyphenyl, guaiacyl, or syringyl, respectively) from which it originates.

Compound	Percentage of $[C_2mim][OAc]$	Treatment time (min)						
		15	45	120	180	240	300	360
trans-Cinnamic acid (H)	0 %	0.00	0.00	0.04	0.06	0.06	0.02	0.00
	10 %	0.00	0.02	0.04	0.02	0.02	0.02	0.02
	70 %	0.02	0.00	0.00	0.00	0.00	0.00	0.00
p-Coumaric acid (H)	0 %	0.00	0.00	0.18	0.24	0.24	0.18	0.18
	10 %	0.00	0.00	0.18	0.28	0.28	0.18	0.18
	70 %	0.54	0.54	0.54	0.54	0.54	0.54	0.54
Epicatechin (G)	0 %	0.00	0.00	0.00	0.00	0.00	0.46	0.42
	10 %	0.00	0.00	0.56	0.46	0.46	0.56	0.46
	70 %	1.24	1.26	1.36	1.36	1.36	1.34	1.36
Guaiacol (G)	0 %	0.26	0.38	0.76	0.61	0.60	0.58	0.54
	10 %	0.22	0.50	1.06	0.96	0.91	0.91	0.91
	70 %	1.06	1.12	1.11	1.10	1.09	1.12	1.25
Isoeugenol (G)	0 %	0.00	0.00	0.06	0.06	0.06	0.06	0.08
	10 %	0.06	0.06	0.06	0.06	0.06	0.06	0.06
	70 %	0.18	0.16	0.16	0.14	0.14	0.16	0.16
Naringenin (H)	0 %	0.00	0.00	0.00	0.00	0.00	0.00	0.00
	10 %	0.00	0.06	0.00	0.00	0.00	0.00	0.06
	70 %	0.36	0.38	0.38	0.40	0.40	0.42	0.42
Quercetin dehydrate (S)	0 %	0.04	0.02	0.08	0.10	0.10	0.12	0.12
	10 %	0.04	0.02	0.08	0.08	0.08	0.10	0.10
	70 %	0.22	0.20	0.22	0.24	0.24	0.26	0.26
Syringaldehyde (S)	0 %	0.00	0.00	0.00	0.00	0.00	0.00	0.00
	10 %	0.00	0.00	0.00	0.04	0.04	0.04	0.04
	70 %	0.00	0.00	0.00	0.00	0.00	0.00	0.00
Vanillin (G)	0 %	0.34	0.52	1.36	1.38	1.38	1.42	1.30
	10 %	0.74	1.26	2.24	2.26	2.26	2.30	2.21
	70 %	2.94	2.96	3.02	3.40	3.40	3.38	3.28

2D ^1H-^{13}C HSQC NMR data of raw Indulin AT and the recovered solid samples

Table S2. Area ratios of integrated regions in the ^1H-^{13}C HSQC NMR spectra of Figure 2 in the Main Manuscript and Figures S4 and S5 below.

Ratio*	Raw Indulin AT	Treatment					
		0 % IL	0 % IL, UV, TiO_2	0 % IL, UV, TiO_2, H_2O_2	70 % IL	70 % IL, UV, TiO_2	70 % IL, UV, TiO_2, H_2O_2
A_γ / C_β	0.11	0.10	0.10	0.09	0.10	0.10	0.10
C_γ / C_β	0.04	0.03	0.03	0.03	0.03	0.03	0.02
B_γ / C_β	0.04	0.02	0.02	0.03	0.02	0.02	0.02
$(A_\alpha + A'_\alpha) / C_\beta$	0.02	0.03	0.02	0.03	0.02	0.02	0.02
$(G_2 + G_5 + G_6) / C_\beta$	0.78	0.77	0.71	0.68	0.58	0.55	0.33
FA_2 / C_β	0.01	0.01	0.01	0.01	0.01	0.02	0.04

* The notation used for the different areas of interest can be consulted in Figure 2 in the Main Manuscript.

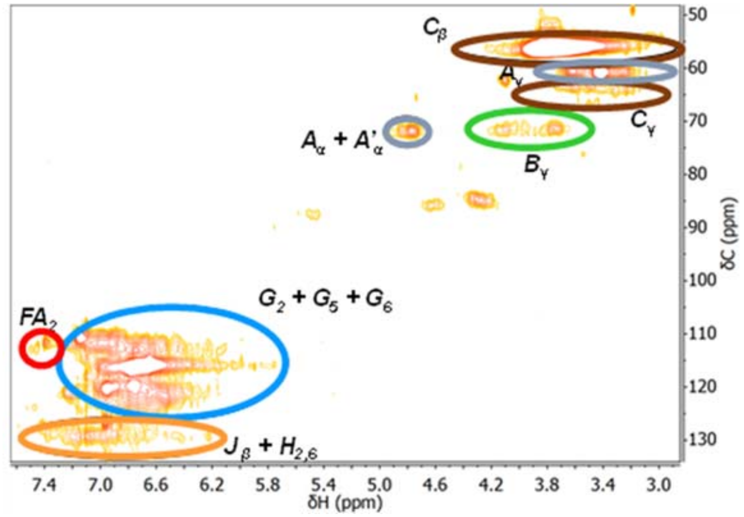

Figure S4. 2D ^1H-^{13}C HSQC NMR spectrum of raw Indulin AT. The colored ellipses in the spectra indicate signals associated with the different generic structures shown in the bottom part of Figure 2 in the Main Manuscript, with frames of the same color.

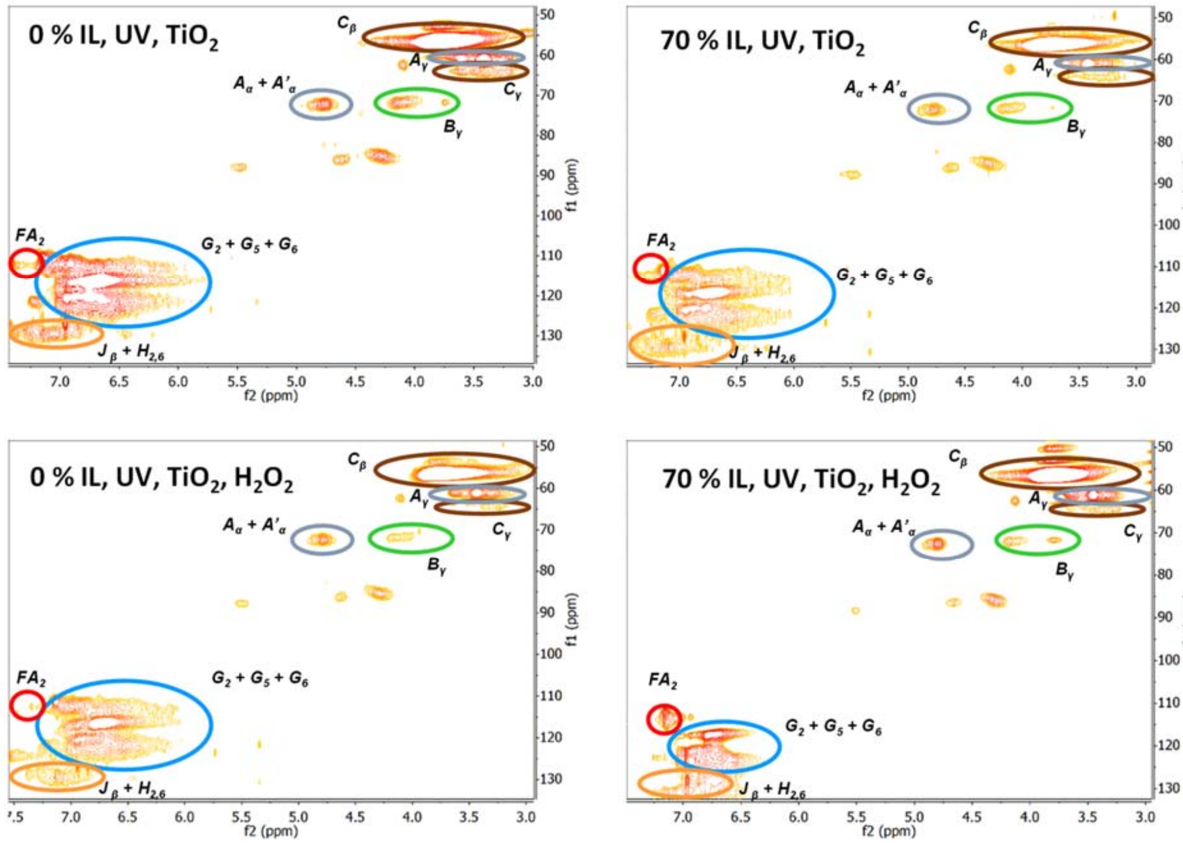

Figure S5. 2D ^1H-^{13}C HSQC NMR spectra of solid samples recovered after 6-hour treatments with water ("0 % IL") or with a 70 % solution of [C_2mim][OAc] ("70 % IL") involving the assistance by UV irradiation with TiO_2 nanoparticles, optionally pre-activated with H_2O_2, as noted in the labels in the upper left corner of each plot. The colored ellipses in the spectra indicate signals associated with the different generic structures shown in the bottom part of Figure 2 in the Main Manuscript, with frames of the same color.

TGA and DSC thermograms

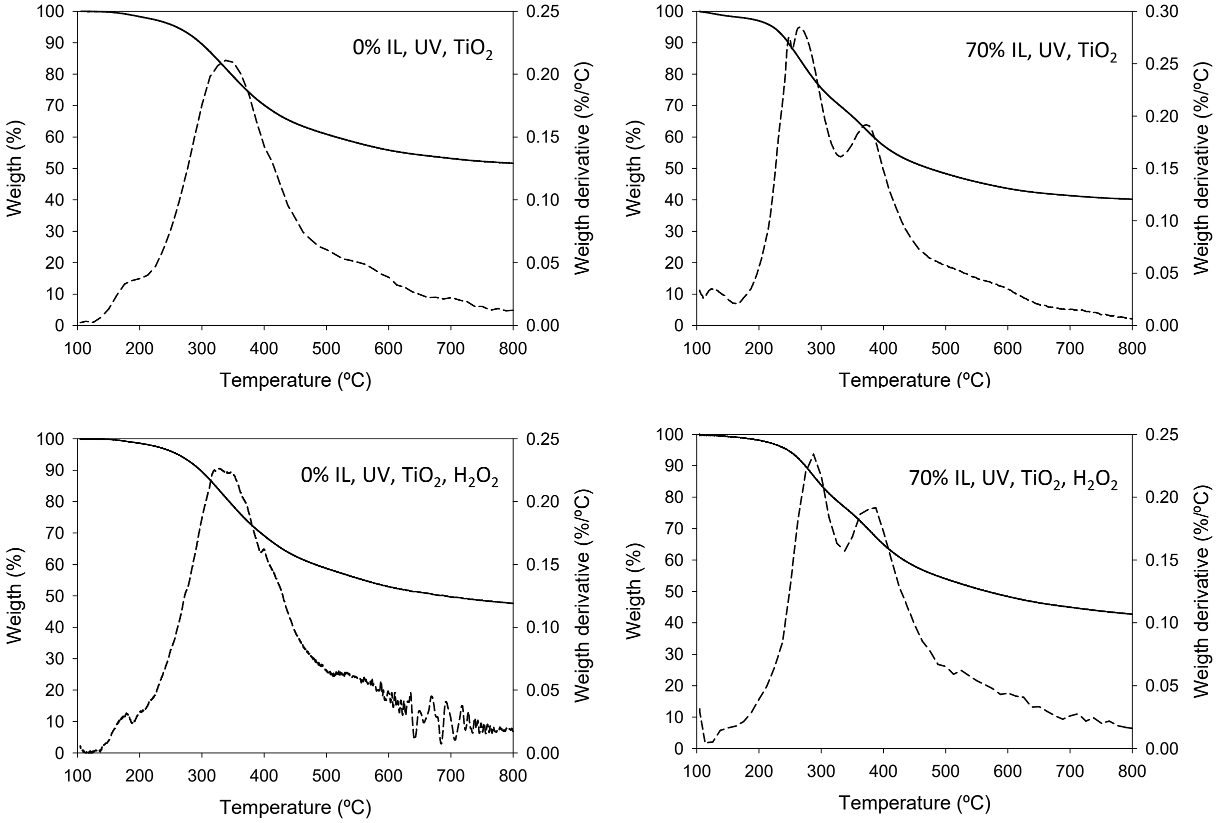

Figure S6. TGA thermograms (solid lines), and the corresponding derivative curves (dashed lines) for the solid samples recovered after 6-hour treatments using water ("0 % IL") or a 70 % aqueous solution of ionic liquid ("70 % IL") as treatment fluids, with the assistance of UV irradiation with TiO_2 nanoparticles, optionally pre-activated with H_2O_2, as noted in the labels in the upper right corner of each plot.

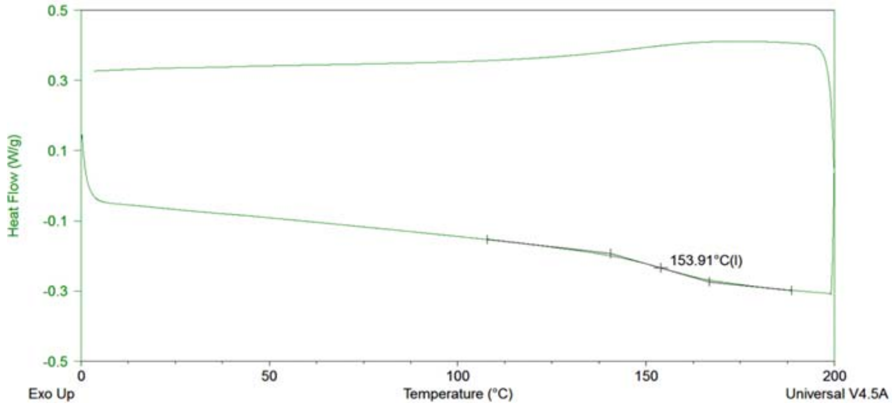

Figure S7. DSC thermogram of raw Indulin AT.

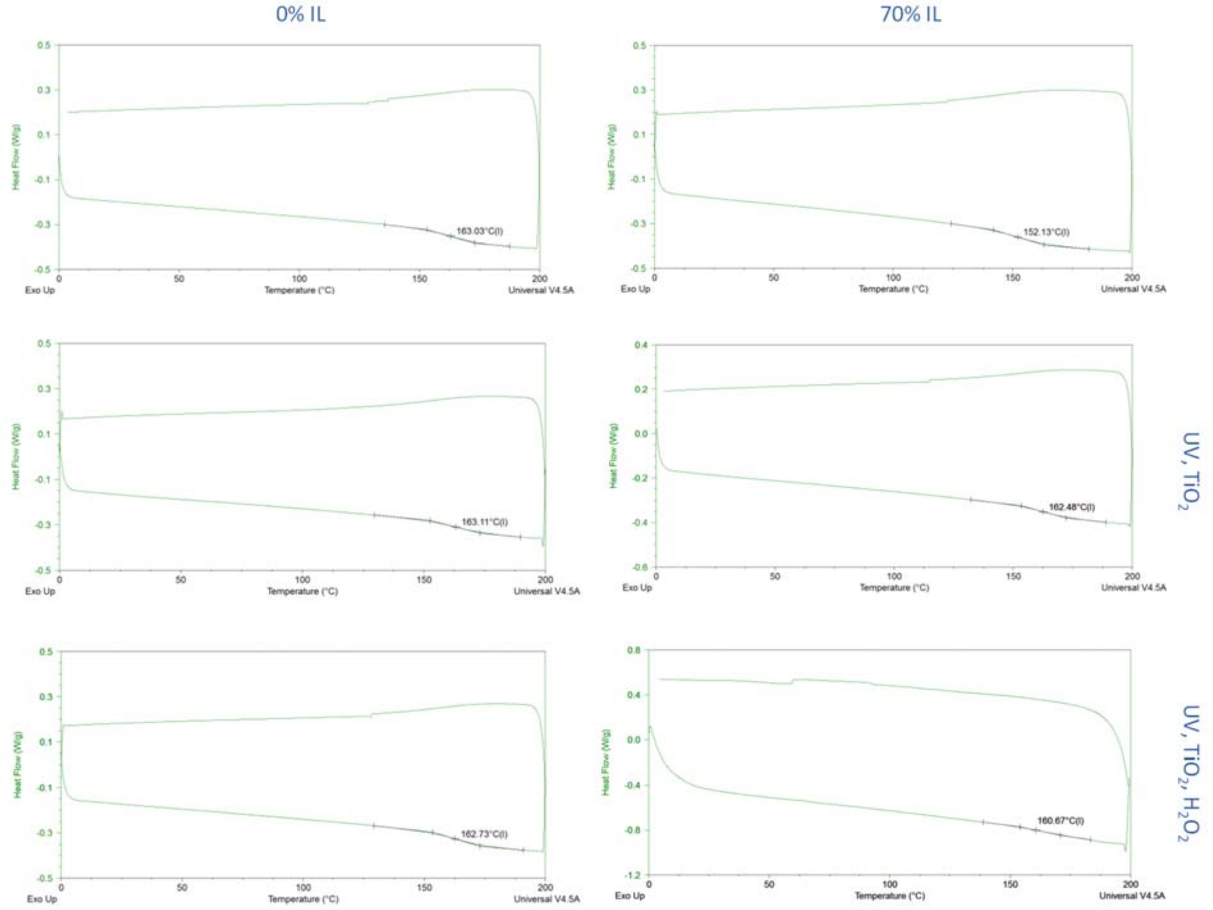

Figure S8. DSC thermograms of the solid samples recovered after 6-hour treatments with water ("0 % IL", plots on the left hand side) or a 70 % solution of the ionic liquid ("70 % IL", plots on the right hand side), with the optional assistance of UV irradiation plus TiO_2 nanoparticles (plots in the middle row) or UV irradiation plus TiO_2 nanoparticles pre-activated with H_2O_2 (plots in the bottom row).
